# Supplementary figures and images for: ECM-mediated positional cues are able to induce pattern, but not new positional information, during axolotl limb regeneration
Source: PLoS One. 2021 Mar 5;16(3):e0248051. doi: 10.1371/journal.pone.0248051 (PMC7935289; doi:10.1371/journal.pone.0248051)

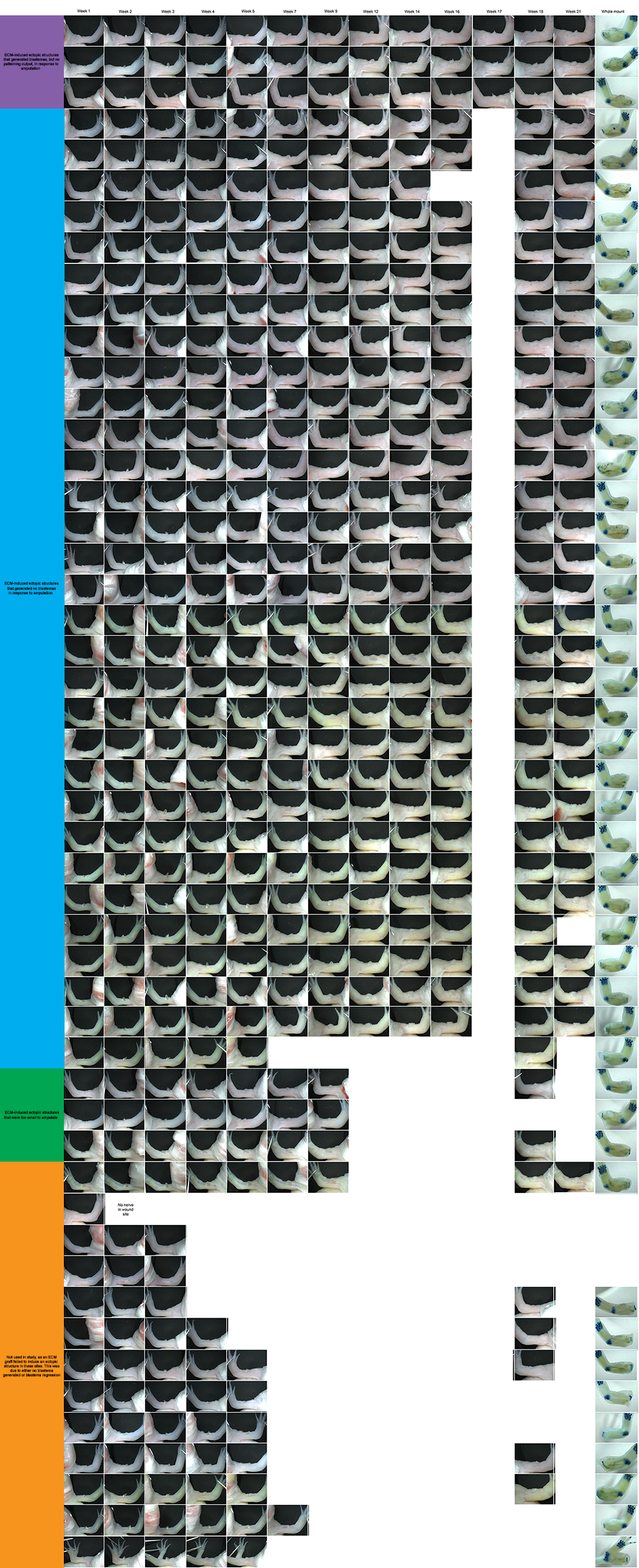

Supplement: S1 Fig — Ectopic structures were induced by grafting decellularized ECM into anterior, innervated wound sites on axolotl forelimbs. Of the 50 anterior ALM sites implanted with posterior derived ECM, live-assessment showed 37 with small, rounded or digit like outgrowths by week 9. At week 13, 34 of the ectopic growths were large enough to amputate and these structures were allowed to regenerate. Amputated structures were monitored for 8 weeks for a regenerative response. 3 of the 34 amputated structures exhibited the formation of a blastema but resulted in no regenerated pattern. The remaining 31 amputated structures simply healed over. Once regeneration/healing was completed, limbs were collected and underwent whole mount cartilage staining. (TIF) [file pone.0248051.s001.tif]

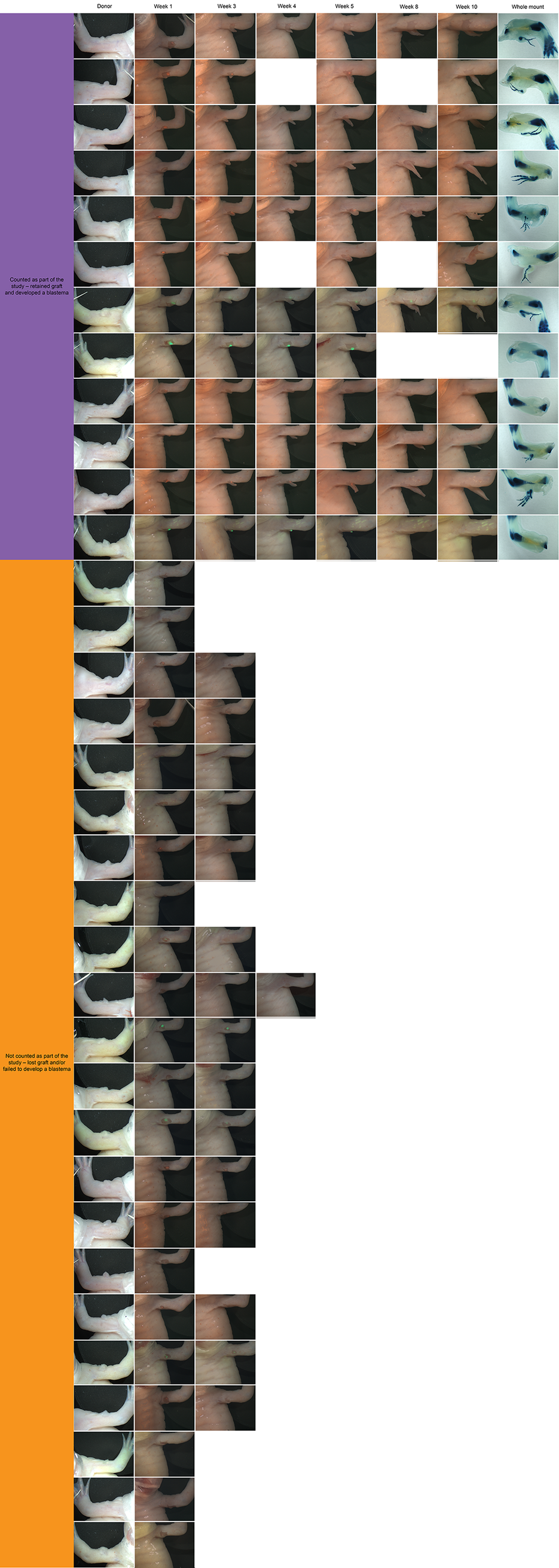

Supplement: S2 Fig — Grafts, derived from ECM-induced ectopic structures, into posterior located ALMs resulted in the generation of complex pattern (9 out of 12). For statistics relating to patterning phenotypes see Table 1. Red and green signal in all live images represents grafted RFP and GFP tissue respectively. Only ALMs that generated a blastema and retained a tissue graft at 3 weeks post-surgery were used for analysis in this study. Once regeneration was completed, limbs were collected and underwent whole mount cartilage staining. (TIF) [file pone.0248051.s002.tif]

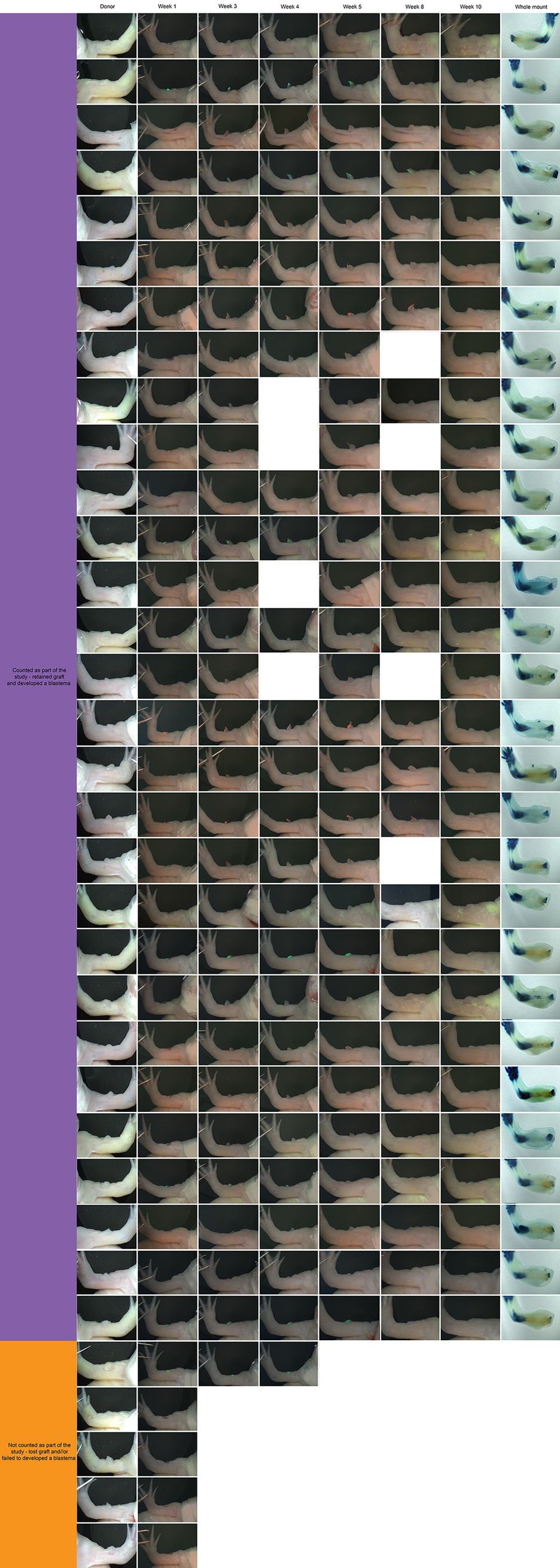

Supplement: S3 Fig — Grafts, derived from ECM-induced ectopic structures, into anterior located ALMs frequently resulted in no ectopic growth (25 out of 29), but sometimes resulted in the generation of a single cartilage nodule (4 out of 29). For statistics relating to patterning phenotypes see Table 1. Red and green signal in all live images represents grafted RFP and GFP tissue respectively. Only ALMs that generated a blastema and retained a tissue graft at 3 weeks post-surgery were used for analysis in this study. Once regeneration was completed, limbs were collected and underwent whole mount cartilage staining. (TIF) [file pone.0248051.s003.tif]
